# Constructing TheKeep.Ca With Thrivers of Cancer in Manitoba, Canada, in Support of Enhancing Patient Engagement: Protocol for a Pragmatic Multimethods Study

**DOI:** 10.2196/63597

**Published:** 2025-01-29

**Authors:** Maclean Thiessen, Kellie Jewitt, Raina Stromberg, Janelle Marie Lamontagne, Genevieve Richardson Tanguay, Annette Albo, Chantale Thurston, Diana E McMillan

**Affiliations:** 1 Section of Medical Oncology and Hematology/Department of Internal Medicine Rady Faculty of Health Sciences University of Manitoba Winnipeg, MB Canada; 2 CancerCare Manitoba Winnipeg, MB Canada; 3 Patient Advisor CancerCare Manitoba Winnipeg, MB Canada; 4 College of Nursing Rady Faculty of Health Sciences University of Manitoba Winnipeg, MB Canada; 5 Health Sciences Center Winnipeg, MB Canada

**Keywords:** patient engagement, patient empowerment, translational research, patient recruitment, development and research, protocol, Manitoba, Canada, cancer, patient advisor, website, research platform, thematic analysis, semi-structured interview, online infrastructure

## Abstract

**Background:**

TheKeep.Ca was built to facilitate engagement with those experiencing cancer in Manitoba, Canada. Constructed between 2020 and 2024 with a group of patient advisors, the website includes information on engagement activities including research participation, the patient advisor role, and how those experiencing cancer can access these Manitoba activities. A link allows visitors to register to be contacted about activities that match their demographics, cancer history, and activity preferences. After TheKeep.Ca was constructed, this protocol was developed to establish TheKeep.Ca as a platform for scientific research focused on optimally engaging those experiencing cancer.

**Objective:**

We asked the following questions: (1) What was the patient advisors’ experience who participated in developing TheKeep.Ca? (2) What are the baseline characteristics of website traffic and registrants at TheKeep.Ca? (3) How does registering with TheKeep.Ca impact the cancer experience?

**Methods:**

The planned launch date for the website and initiation of research activities is January 2025. For objective 1, the active patient advisors (N=6) participating in the website project will be invited to participate in project activities including with responses to a question prompt sheet, semistructured audio-recorded interviews, or both. Responses and interviews will be analyzed using reflexive thematic analysis to understand and inform practices for patient engagement on projects. At the website launch, TheKeep.Ca will become publicly accessible and indexable on internet search engines, but no additional promotional interventions will take place in the initial 6 months resulting in visitors primarily from web search traffic. For objective 2, Google Analytics and website registrant data collected during the first six months will be analyzed to obtain baseline characteristics of website visitors. For objective 3, an online survey will be emailed to registrants six months after the website launch characterizing their website experience, the activities they participated in, and collecting feedback on the website. For objectives 2 and 3, quantitative data will be analyzed using both descriptive and inferential statistics, and qualitative data from open-ended questions will be analyzed using thematic analysis guided by an inductive descriptive semantic approach.

**Results:**

This study was approved by the University of Manitoba Health Research Ethics Board on December 12, 2024 (HS26614-H2024L263). Institutional approval from CancerCare Manitoba is pending as of December 23, 2024. Findings from objective 1 are expected to be finalized within the first six months after the website launch. Those from objectives 2 and 3 are expected by the 12-month mark. Reporting will include peer-reviewed journals, conferences, and a lay-language summary on TheKeep.Ca.

**Conclusions:**

The research outlined in this protocol will facilitate understanding patient advisors’ experience in developing TheKeep.Ca. It will also characterize the website’ effectiveness and its impact on the cancer experience, providing a baseline and direction for future research and development.

**International Registered Report Identifier (IRRID):**

PRR1-10.2196/63597

## Introduction

### Background

The experience of living with cancer is not uniform, and not limited to patients currently receiving treatment. Comprehensive definitions of survivors of cancer include not only those receiving active treatment, those diagnosed and treated in the past but also the friends and family that support them [1]. Similarly, as a leading cause of morbidity and mortality for the global community, cancer does not selectively impact a specific population defined by race, lifestyle, or geographic location.

Because cancer can, and does, impact every human community, engaging with those impacted by cancer, in a way that seeks to understand how cancer services can be improved to provide optimal biomedical care in a way that is respectful, equitable, and supportive of the diverse lifestyles and identities of those living with cancer is essential for the promotion of ethical human care. Nonclinical trial research and work designed to obtain feedback from those with lived experience (eg, such as patient advisors) on service delivery initiatives are two ways that health care professionals can learn how cancer services can be developed to support better the diverse community of those impacted by cancer. However, identifying survivors of cancer to participate in such work can be challenging.

This project started with the goal of creating a website for connecting those experiencing cancer in Manitoba, Canada, with opportunities to improve the cancer journey for others. The website would allow interested survivors of cancer (1) the opportunity to join a database that would facilitate their voluntary participation by notifying them about emerging patient engagement opportunities in Manitoba such as nonclinical trials research or to function as patient advisors. Such a website would facilitate engagement with the Manitoba population with cancer without impacting other existing programs, including clinical service delivery, in the province. The website would be a platform used to study how to better recruit individuals for cancer research; (2) and a tool for rigorously exploring how online approaches can be used to overcome challenges to achieving representative engagement with Manitoba’s diverse population with cancer that includes, but are not limited to, Indigenous peoples, recent immigrants, and those living in urban, rural, and remote communities.

### Understanding Barriers to Recruitment for Nonclinical Trials Research

Language barriers, lack of infrastructure, costs related to advertising and supporting accessibility, and the functional capacity of participants living with illness are just some of the barriers that can impact the recruitment of representative samples for all types of research work [2,3]. In contrast to clinical trial research, which is generally integrated into the participants’ health care, conducted by well-established teams with dedicated infrastructure to support participant recruitment, and comes with the incentive of possibly receiving an experimental intervention that may be superior to the standard of care [2], nonclinical trials researchers face unique challenges. Nonclinical research, such as qualitative and quantitative survey research, as well as longitudinal observational studies, often function with less infrastructure support, clear benefit to the participant, and require commitments on behalf of the participant outside of their responsibilities as recipients of clinical care [4].

Additionally, nonclinical trial studies are often “one-offs” undertaken by trainees, such as graduate and postgraduate researchers, as part of the completion of educational programs. Those responsible for the success of such projects (ie, the trainee) may have less access to gatekept patient populations and other supportive infrastructure, such as research nurses [2]. Unfortunately, these barriers likely result in fewer high-quality research projects, ultimately involving smaller, nonrepresentative samples, and delays in project completion—negatively impacting the quality and diversity of the scientific literature, accessibility to potential participants who may find benefit in participating in such research projects, and the professional trajectory of talented researchers.

### Exploring Barriers to Patient Engagement

Outside of the research recruitment setting, for health care teams, including clinicians, researchers, and administrators, being able to connect with a diverse group of individuals with lived illness experience is becoming increasingly recognized as valuable for program development and improvement [5]. Engagement with individuals functioning as patient advisors to inform project planning, research design [6,7], and infrastructure development is increasingly recognized as an essential process for evolving patient-centered care [5,8,9]. However, as an area of research, understanding how to effectively identify, recruit, and engage with individuals is still evolving [5,10,11]. For instance, it has been suggested that patient advisors may be more likely to be individuals that are passionate about engaging with the health care system than the average patient, resulting in the population of individuals that is engaged with patient advisory work being less likely to be representative of the general patient population [8].

Important work is being carried out to explore how to create better and more meaningful patient engagement opportunities. For instance, aspects of patient engagement such as the timing of activities concerning project lifecycle [6], mitigating power imbalances [11,12], and evaluating outcomes of patient engagement [9] have an evolving body of literature in the spirit of moving from passive to authentic patient engagement [5,9,10,12]. However, questions related to recruitment for engagement appear to be less robustly addressed. A recent systematic review including 142 studies explored patient engagement as a part of research [9]. While the review did identify several different ways that participants were identified, including internet postings, paper advertisements, and invitations from clinicians, studies involving a specific analysis of the impact of different approaches on recruitment effectiveness were not found [9]. As identification of individuals for patient advisory work is often not embedded in the clinical practices of frontline care providers, it is likely that many of the same challenges to recruitment that face nonclinical trial research exist for patient engagement. Work exploring which recruitment approaches for patient engagement are most effective, and how these approaches enable engagement with different populations is important.

### Using Online Infrastructure to Optimize Engagement

Using physical spaces primarily intended for clinical service delivery presents challenges for meaningfully engaging with patients and their friends and family about topics not directly related to patient care. In these clinical areas, such as in ambulatory oncology clinics, patients and their accompanying friends and family members may be already overwhelmed with information regarding their disease and its management [13]. On the other hand, online web spaces can be more easily purpose-built around the needs and interests of those experiencing specific aspects of an illness journey and can be accessed after clinical visits have concluded, during times convenient to the individual [13]. Importantly, the population with cancer is well documented to rely heavily on online information as part of the cancer journey, with some studies demonstrating daily rates of internet use related to cancer exceeding 80% [14]. If carefully developed to meet the needs of those living with cancer, online spaces may be effective at supporting engagement between those living with cancer and health care professionals working to evolve the cancer journey.

### Research Objectives

This protocol outlines how a website intended to support and provide meaningful patient engagement will be studied to advance the science of effective engagement with those living with cancer. As outlined below, TheKeep.Ca website was developed through collaboration with a team of dedicated patient advisors with the aim of creating a webspace to facilitate connecting researchers and health care teams with those who have lived cancer experience as well as providing meaningful resources for those living with cancer in Manitoba and beyond. In addition to helping TheKeep.Ca evolve to better serve the needs of the cancer community in Manitoba, the outcomes from exploring the research objectives outlined in this protocol are intended to provide useful guidance for those intending to embark on pragmatic longitudinal patient engagement projects, such as the development of patient-centric websites, and contribute meaningful findings to the scientific literature regarding how to best engage, online, with those experiencing cancer.

### Research Questions

1. What was the experience of the patient advisors who participated in the development of TheKeep.Ca?
2. How effective is TheKeep.Ca at engaging with the population with cancer in Manitoba in the six months after going live?
  What are the characteristics of website visitors?
  What is the conversion rate from unique website visitors to website registrants?
  What are the characteristics of registrants on the website database?
3. How do the patient engagement activities that TheKeep.Ca facilitate impact the cancer experience for website registrants?
  How does participating in engagement activities promoted by TheKeep.Ca impact the cancer experience?

## Methods

### Building TheKeep.Ca

**Figure 1:**
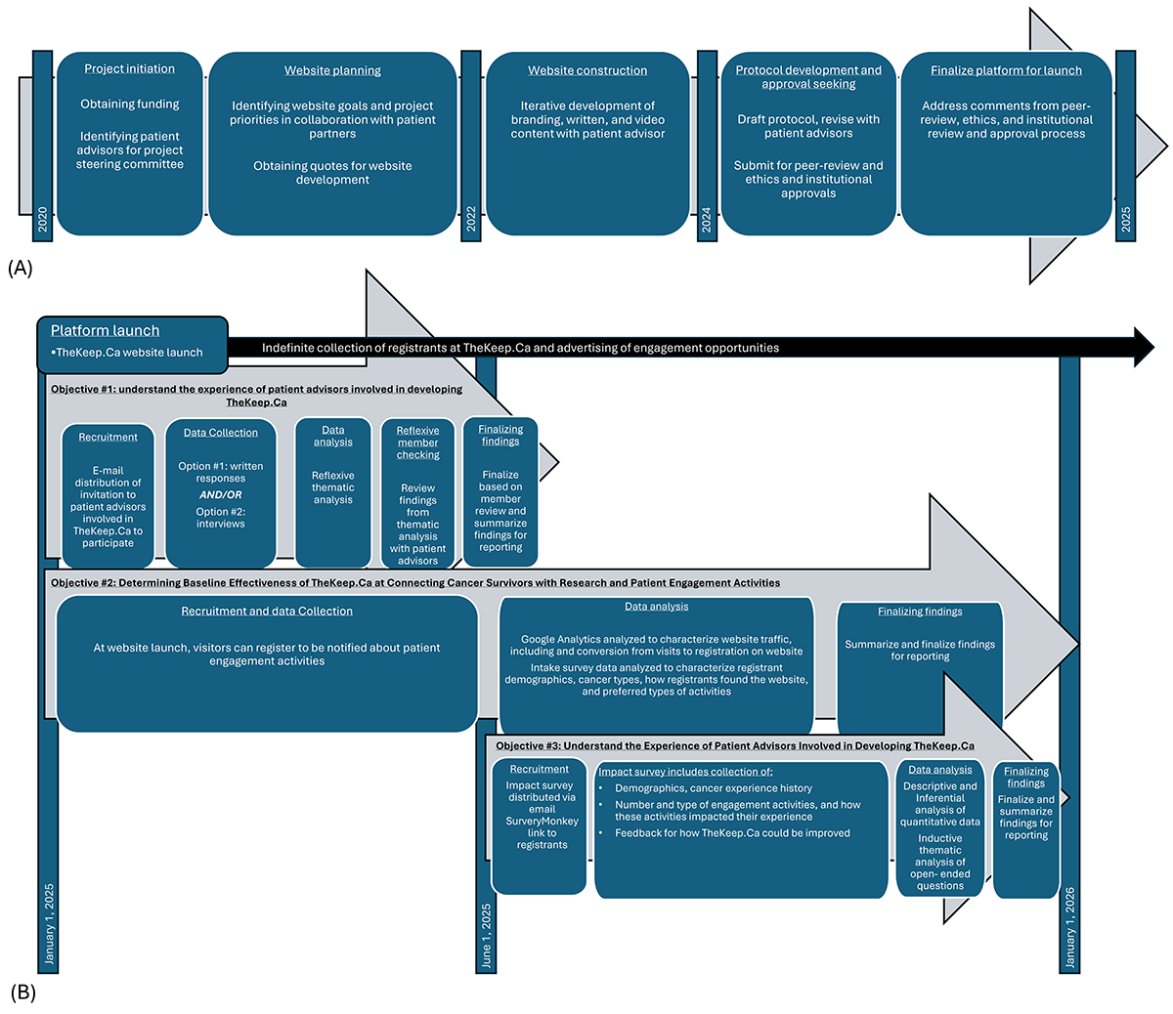
Development of TheKeep.Ca and proposed research activities. (A) TheKeep.Ca was developed over 5 years with a group of patient partners beginning in 2020. Patient partners informed goals for the website, content development, research priorities, and methods. (B) In the six months following the website launch, the patient advisors’ experience of being involved in TheKeep.Ca project will be explored using a qualitative approach. Six months following the launch, data from the registration surveys and Google Analytics will be used to characterize the website’s effectiveness in engaging with the survivors of cancer population in Manitoba. A survey exploring the impact of registration on the TheKeep.Ca website will also be deployed at the 6-month mark with the results used to address the impact of the website on the cancer experience.

Before initiating research activities, TheKeep.Ca website was developed over 4 years through several steps (Figure 1A). After funding had been obtained for the project, patient advisors, including past and present patients with cancer at CancerCare Manitoba (CCMB), were invited to join a patient steering committee to guide the project that was facilitated by the lead author (MT). The patient advisors were recruited both through the CCMB patient advisor program and by psychosocial staff at CCMB.

Through videoconferences, emails, and an online discussion board, the priorities for the website were identified by the steering committee. These included items to satisfy the requirements of the secured funding (ie, a website to inform website visitors about the role of being a patient advisor, a research participant in nonclinical trials research, and a method to populate a database of individuals interested in participating in these roles). Additionally, while the patient advisors identified several important components that could be added, developing a resource directory of supportive care for cancer resources to be hosted on the website curated by patients was selected as the initial patient advisor-championed project to be included on the website.

After the steering committee was formed and the key components were identified, the website was developed in coordination with a professional media design firm (Argyle Media Inc). Facilitated by the lead author (MT), the patient advisors on the steering committee participated in all aspects of developing the website branding, including providing both initial ideas and iterative feedback to the media firm for the logo and site design. Additionally, the patient advisors also participated in content creation for the website, including working alongside a freelance videographer and 2 science communication writers.

In total, 10 current or former patients were involved in the project, however, 4 left the project during its course for reasons including, but not limited to, competing priorities and changes in health status.

### Studying TheKeep.Ca

Once the website was completed, this protocol was developed. Feedback was obtained from the patient advisor steering committee regarding what research objectives they considered important to include regarding the value of a patient engagement website. The protocol was developed by the lead author (MT) and last author (DEM), and then reviewed by the patient advisors, with revisions to this study’s tools and methods made in response to their feedback before submission for peer review.

A schematic outlining the timeline for the activities described in this protocol is presented in Figure 1, including the development of TheKeep.Ca (Figure 1A) and the research activities proposed in this protocol (Figure 1B).

### Research Objective 1: What Was the Experience of Participating in Developing TheKeep.Ca?

The patient advisors who continue to participate in the development of TheKeep.ca (N=6) through membership in the patient engagement steering committee will be invited to share their experience of partnering in the development of TheKeep.Ca. This will be completed within the context of a research study as a voluntary activity. Those who are interested in participating will be invited to contact the last author (DEM) of this protocol, who will obtain informed consent (Multimedia Appendix 1 for the consent form). They will then receive a short intake questionnaire to collect demographic information and details about their cancer experience (Multimedia Appendix 2).

Those who express interest in providing a voluntary written sample following the informed consent process will be provided with a question prompt to provide open-ended written responses (Multimedia Appendix 3). For those who consent to participate in a voluntary semistructured interview, an audio-recorded semistructured interview (estimated duration 60 to 90 minutes) will be conducted following the collection of all written responses. The semistructured interviews will be guided by an interview guide (Multimedia Appendix 4). The question prompts (ie, Multimedia Appendices 1 and 2) were developed by authors MT and DEM, based on feedback MT had received from the patient advisors on the steering committee involved in the project, and reviewed and revised by the steering committee before finalizing this protocol. Transcripts of the audio recordings will be prepared using a professional transcription service (ie, Scribie), and both the written survey responses and interview transcripts will be deidentified before analysis.

Reflexive thematic analysis (RTA) [15] will be used to analyze the data collected. RTA is a nuanced iteration of the thematic analysis approach initially described by Braun and Clarke [16]. Specifically, RTA explicitly addresses several methodological questions that have been identified since the initial publication describing thematic analysis by Braun and Clarke [16]. Specifically, RTA is not considered a positivistic, descriptive, rigid methodology. Instead, it embraces constructivism, an inductive flexible approach to data analysis, consideration of latent meaning, and an emphasis on the interpretation of the data by the researcher [15,17]. Compared to other iterations of thematic analysis, RTA was chosen because the experience of the patient advisors involved in the development of TheKeep.Ca was thought to be complex, with many aspects likely to be unknown a priori to the researchers, requiring more than a descriptive summary of the data collected to provide a meaningful exploration and contribution to the literature.

The steps for data analysis will be in keeping with those described for RTA [15-17], progressing in a relatively linear manner through six phases including (1) familiarization with the data once data collection is completed, (2) generating initial codes using line by line coding, (3) generating themes, (4) reviewing potential themes, (5) defining and naming themes, and (6) producing the report. In keeping with a nonpositivistic stance, the volunteers from the steering committee will be invited to provide member reflections in a group setting as part of phase 6 once a preliminary version of the report is ready. The purpose of this activity will be to “explore gaps in understanding … and consider how to acknowledge and present these in the written report” [15], as opposed to validating findings. The final report will be revised to reflect the findings from this final group activity.

### Rigor and Trustworthiness

The trustworthiness of the research findings will be facilitated by ensuring practices that enhance credibility, transferability, dependability, and confirmability [18,19]. Prolonged engagement with this study’s participants and data, as well as a review of study findings with participants before finalizing study results, are techniques that will be used to address the credibility of the findings. By providing a rich description of the data, the contexts in which it was collected, and the resulting findings, as well as by considering both latent and semantic themes, the transferability of this study’s findings to other contexts related to patient engagement will be supported. Dependability will be supported by recording written field notes in addition to the audio recording of semistructured interviews, the keeping of a reflexive journal to document the processes and decisions related to the generation of codes during data analysis, and the use of NVivo (Lumivero LLC, version 13, released 2020) software which provides an auditable record of the steps by which the data was coded. Conformability, or whether the research findings follow from the data collected, will be supported through the techniques described in support of credibility, transferability, and dependability [18]. Additionally, conformability will be further supported by recording additional aspects of the research process in the reflexivity journal, beyond the specific decisions and processes related to the development of the codes. For instance, it is anticipated the DEM will record information about the daily experience of completing the data collection and analysis, as well as reflections on their personal experience with the research [18,19], in support of connecting personal experience with conducting research with the data and generated findings.

Reporting of the findings will conform with COREQ (Consolidated Criteria for Reporting Qualitative Research) guidelines to facilitate rigorous reporting [20]. In keeping with this, in addition to thorough reporting of study methods and findings reflecting the checklist items, a reflexivity statement will be included in the final report which will include, but not be limited to, a characterization of DEM’s professional identity, and personal biases, assumptions, and reasons and interests in the research topic.

### Research Objective 2: What Is the Baseline Effectiveness of TheKeep.Ca at Connecting Survivors of Cancer With Research and Patient Advisor Activities?

Data from website visitor metrics and registrant data collected over the first 6 months after the website goes live will be used to establish a baseline of the effectiveness of TheKeep.Ca at recruiting members of the Manitoba population with cancer for engagement activities. Google Analytics will be used to provide metrics related to user activity on the website. The methodology for using Google Analytics to understand website traffic is informed by that used by previous authors [21]. Specific metrics to be used will include, but not be limited to, total users, type of devices used, geographic locations of users, session length, websites’ pages viewed, number of page views per page, bounce rate (ie, number of users leaving the site without viewing additional pages), site entrances, and page interactions.

Data from registrants on the TheKeep.Ca engagement database will be used to further characterize the effectiveness of the website, as well as the population that it effectively engages within the first six months after going live. Specifically, respondent demographics, information about their cancer experience, preferred types of activities, and how they found the website are tracked as part of the registration survey (Multimedia Appendix 5).

Data analysis will include the generation of descriptive statistics of the website metrics and registrant characteristics. Descriptive statistics will be generated from both the Google Analytics data as well as the registrant data. Inferential statistics, including chi-square and *t* test (2-tailed), will be used to describe differences in Google Analytics metrics between website visitors from Manitoba and outside of Manitoba, as well as by device. Additionally, inferential statistics will be used to describe differences in disease characteristics and engagement preferences by registrant demographics, including age, gender, and location. Open-ended question data will be summarized using a descriptive semantic inductive approach to thematic analysis [15,16], with the aim of providing a simple summary of open-ended question data.

### Research Objective 3: How Does Registration on TheKeep.Ca Impact the Cancer Experience?

After the initial 6 months after TheKeep.Ca has gone live, an online SurveyMonkey survey (Multimedia Appendix 6 for the survey) will be distributed to the website registrants who consented to be contacted with a follow-up survey as part of the initial registration questionnaire. Demographic data and information about the registrant’s cancer experience (ie, date of diagnosis, type of diagnosis, patient vs informal caregiver, and treatment intent) will be captured, as well as the number and type of engagement activities they have participated in that were advertised on TheKeep.Ca. Using a combination of Likert-style responses and open-ended questions the survey will evaluate the experience of being a registrant on TheKeep.Ca, the effectiveness of TheKeep.Ca in connecting individuals with engagement opportunities, the impact of engagement activities on the experience of registrants, and identify ways that TheKeep.Ca can provide a better experience to website registrants. Descriptive statistics will be generated to describe the respondent population and the overall experience of the group. Inferential statistics will be used to identify differences between populations, based on demographics and cancer experience, and the impact TheKeep.Ca had on respondents’ cancer experience. Open-ended question data will be summarized using a descriptive semantic inductive approach to thematic analysis [15,16], with the aim of providing a simple summary of open-ended question data.

Development of the research protocol related to objectives 2 and 3 was informed by the CHERRIES (Checklist for Reporting Results of Internet E-Surveys) checklist to facilitate rigorous reporting [22]. For qualitative data, NVivo will be used to assist with qualitative data management and analysis. Quantitative data analysis will be conducted using SPSS (IBM Corp, version 27, released 2020).

### Ethical Considerations

This study was approved by the University of Manitoba Health Research Ethics Board on December 12, 2024 (HS26614-H2024L263). Institutional approval from CCMB is pending as of December 23, 2024. Participants, including both registrants on TheKeep.Ca website as well as the patient advisors involved in the development of this project, did not receive compensation of any kind. Enrollment on TheKeep.Ca website is strictly voluntary, with informed consent being implied as part of the registration process.

All data will be deidentified before analysis. Data related to objective 1 will be linked to a master list, which will be destroyed following the completion of research activities outlined in this protocol. Data related to objective 2 (ie, registrant responses) will be deidentified before analysis for research purposes; however, names and contact information will be required to be retained on this data to facilitate screening and informing registrants of relevant patient engagement activities. However, this data will not be shared with any other party and be available only to the research team (ie, MT and DEM). Data from objective 2 is being collected in an anonymized fashion, with an absence of information that would support the identification of individuals. Notably, while the email addresses provided by registrants on the website will be used to collect data for objective 3, the responses will not be linked to the email addresses or the initial responses provided by the individuals at the time of website registration.

Patient advisor involvement, including participation on the steering committee, being listed as an author on this paper, and participation in the research activities related to objective 1, was strictly voluntary, and would not impact their ability to participate in any aspect of the activities related to this project. The patient advisors listed as authors on this protocol provided expressed written consent to be listed as such and fulfilled the criteria to be listed as authors in peer-reviewed journal publications, including active involvement in all aspects of the project and protocol development. Documentation of this consent was provided to the editor for review as part of this peer-reviewed process.

All images, names, and videos of individual thrivers of cancer presented on TheKeep.Ca website are used with expressed consent. The website does include stock images obtained from a third party (ie, Adobe Stock Images) which are used per the images’ respective licensing agreements.

## Results

TheKeep.ca will go “live” after this study receives institutional approval from CCMB, becoming searchable on major search engines (eg, Google) and accessible to the public. The results related to research objective 1 are expected to be submitted for peer-reviewed publication within 6 months after the website becomes accessible to the public with results related to research objectives 2 and 3 being submitted for peer review by the 12-month mark. In addition to being presented in peer-reviewed manuscripts, the results will also be shared at national and international conferences as well as presented on TheKeep.Ca website in written and video format using layperson language.

## Discussion

### Principal Findings

Addressing the three research objectives outlined in the protocol is important for informing future work in several ways. Regarding research objective 1, better understanding the experience of the patient advisors who were, and continue to be, involved in TheKeep.Ca project is important for evolving how TheKeep.Ca project is conducted in the future, as well as informing how other projects where authentic longitudinal patient engagement is desired. While high-quality reviews [5,9] and frameworks to guide patient engagement activity planning exist [23], such as those available through the Patient-Centered Outcomes Research Institute [24], the findings from this work will provide real-world examples of the experiences, challenges, and lessons learned from a complex project where authentic longitudinal engagement with thrivers of cancer was foundational. Specifically, identifying what parts of this project have been frustrating, stressful, or otherwise not rewarding, for the patient advisors as well as which aspects were viewed as providing a positive and meaningful experience is expected to make a meaningful addition to the literature [10].

Regarding objectives 2 and 3, obtaining baseline data regarding how effective the website is at engaging those living with cancer both inside and outside of Manitoba, as well as the characteristics of the registrants, is important for guiding future work to better engage with the diverse population of individuals living with cancer both inside and outside of Manitoba. It is anticipated that the baseline Google Analytics data will provide meaningful insights per who engages with TheKeep.Ca website. Similarly, the initial registrant experience survey (ie, objective 3) will provide important data about how to enhance the experience of those that register on TheKeep.Ca. This data will reflect the experience of engaging with TheKeep.Ca in the absence of planned social media campaigns and advertising at the cancer center. As the website develops, and campaigns to promote the website are developed, the baseline data will provide a real-world comparison to gauge the effectiveness of promotional interventions such as social media advertising campaigns, paid Google Adwords campaigns, and posters placed in cancer care facilities, or private businesses. This data will be helpful not only for helping TheKeep.Ca evolve into a tool for engaging meaningfully with Manitoba’s population with cancer but also for other groups endeavoring to engage with either specific populations or a representative sample of those impacted by cancer [8].

### Limitations

This protocol has two main limitations. First, to address research objective 1, not all patient advisors who participated in the development of TheKeep.Ca will be able to be invited for interviews. At the time of protocol submission, three of the initial patient advisors involved left the project, for several reasons—not all of which were disclosed. While, from both a scientific and quality improvement perspective, it would be ideal to explore the reasons for dropping out from the project to provide a better experience for those in the future, however, out of respect for the privacy of the individuals that withdrew, this is not possible. Ideally, experience data related to participation in the project would have been captured longitudinally, throughout the project. However, the decision to establish TheKeep.Ca as a research platform was made relatively late in the project history, as such the need to formally collect rigorous experience data was not considered previously.

Second, there is a risk that initial recruitment to the TheKeep.Ca registrant database will be limited, resulting in limited data to address objectives 2 and 3. TheKeep.Ca, as a research platform, represents a novel endeavor, making predicting the initial recruitment rate and the resulting sample size for the first six-month sample size impossible. Additionally, as the goal is to obtain baseline data for traffic and engagement with the site, before additional advertising interventions as outlined above, traffic may be very low. As a result, it is recognized that the generation of meaningful inferential statistics may not be possible. Regardless, the initial data from the first six-month period will serve as an important baseline, essential for understanding the impact of future campaigns to promote the website and recruitment to the website registry.

### Conclusion

The webspace that became TheKeep.Ca was initially proposed as a simple tool to connect those who have experienced cancer firsthand with opportunities to engage with researchers and other teams working to improve the cancer journey in Manitoba, Canada. However, through endeavoring to seek authentic [9] collaboration with a group of patient advisors over several years, the simple proposal evolved into something with the potential to become much more than that. In keeping with the original proposal, TheKeep.Ca is a catalyst, enhancing the capacity for connection between those willing to share their hard-won firsthand knowledge with health care professionals. However, it is also a place where patient advisors can contribute directly to improving the cancer journey, as it provides online infrastructure for patient advisor-led initiatives—such as the curated resource directory. Lastly, through a rigorous research program, beginning with the work outlined in this protocol, TheKeep.Ca project aims to achieve these goals while, at the same time, advancing what is known about patient engagement for the benefit of those living with cancer in Manitoba and beyond.

## Appendix

Multimedia Appendix 1 Consent form for objective 1.

Multimedia Appendix 2 Intake questionnaire for objective 1.

Multimedia Appendix 3 Written prompt sheet for objective 1.

Multimedia Appendix 4 Semistructured interview guide for objective 1.

Multimedia Appendix 5 Registration survey for objective 2.

Multimedia Appendix 6 Six-month follow-up survey for objective 3.

## Abbreviations

CCMB: CancerCare Manitoba
CHERRIES: Checklist for Reporting Results of Internet E-Surveys
COREQ: Consolidated Criteria for Reporting Qualitative Research
RTA: reflexive thematic analysis

## Data Availability

To protect the confidentiality of the research participants, deidentified data, including qualitative data related to objective 1, and survey response data from objectives 2 and 3 will not be made publicly available. However, it may be obtained upon reasonable request to the authors, and subsequent approval of the request after review by the University of Manitoba Health Research Ethics Board.
