# Supplementary material for: Constructing TheKeep.Ca With Thrivers of Cancer in Manitoba, Canada, in Support of Enhancing Patient Engagement: Protocol for a Pragmatic Multimethods Study
Source: JMIR Res Protoc. 2025 Jan 29;14:e63597. doi: 10.2196/63597 (PMC11822311; doi:10.2196/63597)
Supplement: Multimedia Appendix 1 [file resprot_v14i1e63597_app1.docx]

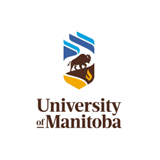


TITLE: Exploring the Experience of Patient Advisors Who Participated in a Collaborative Longitudinal Project Resulting in The Creation of TheKeep.Ca

INVESTIGATORS: Dr. Maclean Thiessen, Dr. Diana McMillan

PRIMARY CONTACT: Dr. Maclean Thiessen

Phone: (204) 237 2472

E-mail: maclean.thiessen@umanitoba.ca

You are being asked to participate in a research study. This form is part of the process of informed consent, it should give you some background on the research study and what your participation will involve. Please take the time to review this consent form and discuss any questions you may have with the study staff, friends or family before you make your decision. If you would like more detail about something mentioned here, or information not included here, please ask the study staff.

BACKGROUND

Engaging with patients and their friends and family to inform the development of infrastructure is increasingly being recognized as a key component of improving the experience. TheKeep.Ca was developed in collaboration with individuals that experience cancer first hand over a number of years. Unlike many projects that involve patient advisors that have clear road map, and engage with patient advisors at specific times, this project was longitudinal, dynamic, and conducted without a clear end point. Understanding the experience of the patient advisors who participated in the project is important, as these types of pragmatic projects may yield excellent results, but should also be conducted in a way that is respectful, and positive for the patient advisors involved. Little is known about the experience of patient advisors who participate in such projects.

PURPOSE OF THE STUDY

The purpose of this study is to understand the experience of the patient advisors who participated in this project, from its initial stages to the completion of TheKeep.Ca website.

 PARTICIPANT SELECTION

The patient advisors who participated in the development of TheKeep.Ca and remain active in the project will be invited to participate in this study. They participated in the development of this protocol, with Dr. Thiessen and Dr. McMillan. They have also been working with Dr. Thiessen in the development TheKeep.Ca.

STUDY PROCEDURES

Participants will:

- Be asked to review and, if interested in participating, complete this informed consent document
- Be invited to complete a written questionnaire
- Be invited to complete a semi-structured interview with Dr. McMillan at a mutually convenient time. Estimated time of the interview is 60 – 90 minutes. The interviews are planned to occur virtually (i.e., MS Teams, or telephone), but in person interviews can be arranged if requested.
- Be invited to review and provide feedback on the findings of the data analysis, once the collected data is de-identified and initial analysis has been conducted.

RISKS and DISCOMFORTS

This study involves reflecting on the experience that you have had following the cancer diagnosis and what factors influenced this experience. There are very few risks, however you may find that thinking about these things can be difficult and upsetting. You do not have to answer any question that makes you feel uncomfortable or that you find too upsetting. You might experience distress as result, and you might find it helpful to seek out emotional and psychological support. These services are available through Patient & Family Support Services at CancerCare Manitoba. They can be reached at 1-866-561-1026 or by emailing:

pfssinquiry@cancercare.mb.ca

BENEFITS

You may find that participating in this study may help you feel better about your experience with cancer, that you are contributing to make making a difference for Manitoban’s living with cancer, you may also find that the activities related to this project assist your work related to patient advising with CCMB or other organizations. However, there is no guarantee that this research will help you. The information we get from this study may help support those living with cancer in the future.

PARTICIPATION

Participation in this study is completely voluntary. Deciding to not participate in this study will not impact your ongoing participation in TheKeep.Ca project, or other aspects of your patient advisor work, or any ongoing clinical care.

Once you have participated in the study your data cannot be removed from the study.

COST or PAYMENT

There is no cost to you to participate in this research study.

Participants are not paid to participate in this research study.

The study team has received no professional fees or personal reimbursement for conducting this study. Funding to cover the costs of conducting study activities has been provided by the CancerCare Manitoba Foundation.

CONFIDENTIALITY

All data collected will be stored on password-protected encrypted USB drives when in use, otherwise it will be stored on secure servers at CancerCare Manitoba. Audio files and subsequent transcripts will be transferred to and from the transcription company using password-protected encrypted USB drives or through the company’s secure portal. Your name or any data that identifies you will be removed from collected data (audio transcriptions and written responses) prior to data analysis. Your identity will be linked to your study data through a master list, which will be stored in a separate file from the collected deidentified data. Results from the study will be presented in a way that it will make it not possible to link you to any specific quote or finding. At the end of the data collection and analysis the master list, audio recordings, and any written data containing identifying data will be destroyed. This is expected to occur in the latter half of 2025. Deidentified data will be retained indefinitely for research and teaching purposes by the researchers, but not shared with other parties unless reasonable request is made after approval by the University of Manitoba Health Research Ethics Board.

SIGNATURES

Your signature on this form indicates that you have understood the information regarding your participation in the research project and agree to participate. In no way does this waive your legal rights nor release the investigators or involved institutions from their legal and professional responsibilities. You are free to withdraw from the study at any time without jeopardizing your health care. If you have further questions concerning matters related to this research, please contact the principal investigator:

Dr. Maclean Thiessen: (204) 237 - 2472

For questions about your rights as a research participant, you may contact The University of Manitoba, Bannatyne Campus Health Research Ethics Board Office at (204) 789-3389.


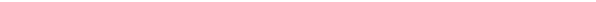


| Participant’s Name | Signature and Date |
| --- | --- |


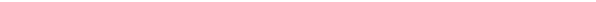


Witness’ Name Signature and Date

The study details and this consent document has been reviewed with the participant by the investigator indicated below. This has been done either over the phone, videocall, or in person.


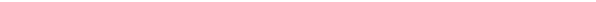


Investigator’s Name Signature and Date

Reason for telephone consent, if applicable:


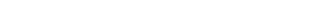


The University of Manitoba Research Ethics Board, which oversees the ethical acceptability of research involving humans, has reviewed and granted ethics approval for this study.

A signed copy of this consent form will be given to you to keep for your records and reference.
