# Supplementary material for: Constructing TheKeep.Ca With Thrivers of Cancer in Manitoba, Canada, in Support of Enhancing Patient Engagement: Protocol for a Pragmatic Multimethods Study
Source: JMIR Res Protoc. 2025 Jan 29;14:e63597. doi: 10.2196/63597 (PMC11822311; doi:10.2196/63597)
Supplement: Multimedia Appendix 2 [file resprot_v14i1e63597_app2.docx]

**Intake Questionnaire**

1. Please complete this survey at the time of completing your consent form. Its purpose is not to exclude individuals, but to ensure a diverse group of people are included in this study.
2. This questionnaire uses the term “living with cancer” to refer to individuals that have either received a diagnosis of cancer, supporting someone that is, or have been affected by someone else's cancer diagnosis.

Year of Birth: Gender:

Marital Status:

| Single | Married/Common Law | Divorced | Widowed |
| --- | --- | --- | --- |

Education Level:

| Some High School | Graduated High School | Some University/College | Graduated University/College |
| --- | --- | --- | --- |

Is English your first language?

| YES | NO |
| --- | --- |

Were you born in Canada?

| YES | NO |
| --- | --- |

If you were not born in Canada, how many years have you lived in Canada:

|  |
| --- |

Annual Household Income:

| Less than $20,000 | $20,000 to $50,000 | $50,000 to $100,000 | More than $100,000 |
| --- | --- | --- | --- |

What kind of cancer do you, or the person you are supporting have? (please fill in the blank)

|  |
| --- |

Is the cancer being treated with curative or non-curative intent?

| Curative | Non-curative | Unsure |
| --- | --- | --- |

Please circle the role that describes you the best?

| Former or Current Cancer Patient | Supporter of a past or present cancer patient | Both |
| --- | --- | --- |

| For Researcher Use Only  Study Number: |
| --- |
