# Supplementary material for: Constructing TheKeep.Ca With Thrivers of Cancer in Manitoba, Canada, in Support of Enhancing Patient Engagement: Protocol for a Pragmatic Multimethods Study
Source: JMIR Res Protoc. 2025 Jan 29;14:e63597. doi: 10.2196/63597 (PMC11822311; doi:10.2196/63597)
Supplement: Multimedia Appendix 3 [file resprot_v14i1e63597_app3.docx]

**Intake Questionnaire**

1. Please complete this survey at the time of completing your consent form. Its purpose is not to exclude individuals, but to ensure a diverse group of people are included in this study.
2. This questionnaire uses the term “living with cancer” to refer to individuals that have either received a diagnosis of cancer, supporting someone that is, or have been affected by someone else's cancer diagnosis.

Year of Birth: Gender:

Marital Status:

| Single | Married/Common Law | Divorced | Widowed |
| --- | --- | --- | --- |

Education Level:

| Some High School | Graduated High School | Some University/College | Graduated University/College |
| --- | --- | --- | --- |

Is English your first language?

| YES | NO |
| --- | --- |

Were you born in Canada?

| YES | NO |
| --- | --- |

If you were not born in Canada, how many years have you lived in Canada:

|  |
| --- |

Annual Household Income:

| Less than $20,000 | $20,000 to $50,000 | $50,000 to $100,000 | More than $100,000 |
| --- | --- | --- | --- |

What kind of cancer do you, or the person you are supporting have? (please fill in the blank)

|  |
| --- |

Is the cancer being treated with curative or non-curative intent?

| Curative | Non-curative | Unsure |
| --- | --- | --- |

Please circle the role that describes you the best?

| Former or Current Cancer Patient | Supporter of a past or present cancer patient | Both |
| --- | --- | --- |

| For Researcher Use Only  Study Number: |
| --- |

**Open Ended Questions (please type as much as needed, or attached additional pages):**

1. Why did you agree to get involved in this project?
2. Were the project objectives clear?
3. Do you feel that the project objectives changed over the course of the project?
4. Has anything been frustrating or challenging about the project? If so, what?
5. How could this project be improved?
6. What things have been rewarding about the project?
7. Do you feel that the outcome, so far, will be valuable?
8. Compared to other patient advisor work you have participated in, what stands out about this project?
9. How did participating in this project impact your experience as a cancer survivor?
10. What do you see for the future of this project?
