# Supplementary material for: Constructing TheKeep.Ca With Thrivers of Cancer in Manitoba, Canada, in Support of Enhancing Patient Engagement: Protocol for a Pragmatic Multimethods Study
Source: JMIR Res Protoc. 2025 Jan 29;14:e63597. doi: 10.2196/63597 (PMC11822311; doi:10.2196/63597)
Supplement: Multimedia Appendix 4 [file resprot_v14i1e63597_app4.docx]

Estimated time: 60 - 90 minutes

Opening statement: Thank you for agreeing to participate in this research interview. It is being recorded as part of a research study exploring the experience of collaborating in the creation of TheKeep.Ca. I will share the recording only with the professional transcription service who has agreed to keep the recording confidential. Any responses you give will have details that could result in your being identified removed prior to being included in the data analysis.

You are free to pause the interview, take a break, or stop altogether at any point. Are you ready to begin?

Semi-structured interview questions:

1. Tell me a little bit about why you participate in work as a patient advisor?
2. What has your experience been in working on this project?
3. How does this project compare to other patient advisor work you have participated in?
4. What aspects have you enjoyed?
5. What aspects have been challenging or not enjoyable?
6. What would you like to see happen in the future with this project?
7. What changes would you like to see in terms of how this project is conducted?
8. Is there anything else you would like to share?

Concluding statement: Thank you for participating in this interview. The next steps will be for me to complete data analysis of the information collected as part of these interviews. When this is finished you, and the others that participated in this project, will have the opportunity to review the findings with the aim of ensuring that you and your colleagues feel that that was is being presented reflects your experience. It will likely take 2-3 months before I am able to follow-up.
