# Supplementary material for: Constructing TheKeep.Ca With Thrivers of Cancer in Manitoba, Canada, in Support of Enhancing Patient Engagement: Protocol for a Pragmatic Multimethods Study
Source: JMIR Res Protoc. 2025 Jan 29;14:e63597. doi: 10.2196/63597 (PMC11822311; doi:10.2196/63597)
Supplement: Multimedia Appendix 5 [file resprot_v14i1e63597_app5.pdf]

Things you need to know before you provide your information...

By completing the following form, you are consenting to join the database maintained by TheKeep.Ca.

The database is intended to connect people who have experienced cancer with opportunities to improve the cancer experience for others through research and patient advisor work. At this time, the opportunities linked to this database are primarily for those in Manitoba.

If you are located outside of the province of Manitoba, or have not received cancer care in Manitoba, you may find that the activities connected with this project that you are eligible for are limited. However, you are still welcome to join the database.

The information you provide helps TheKeep.Ca team understand how to improve the website and connect you with the types of opportunities you are interested in. Please read the description of each opportunity type carefully and only select the ones where you are comfortable with how your information will be handled by TheKeep.Ca.

The form being used is highly secure, compliant with the highest standards of data security. It is unlikely, but possible that a data breach may occur.

The information you provide on the form may contribute to research and quality improvements reports. This will be done in a way where you will not be able to be identified by others.

Providing your information through this survey and registration on the TheKeep.Ca database, should be considered strictly voluntary. Your responses, or decision to not complete this form, will not impact your care or your eligibility to participate in other patient engagement activities including, but not limited to research or patient advisor work, in anyway.

What types of opportunities are you interested in?

Please select the types of opportunities you are interested in. Please read each description carefully to understand how your information will be used by TheKeep.Ca.

1. I would like to receive:

- ☐ **The Navigator: A CCMB e-mail newsletter for patients and informal caregivers.** Your email address will be shared directly with the publishers of the newsletter so they can distribute it to you. The other information you provide in this form will not be shared.
- ☐ **Information about research opportunities that are not specific to a certain cancer experience.** Information about these kinds of opportunities will be shared with you directly by TheKeep.Ca team, your information will not be shared with other groups. For instance, an online survey open to all cancer patients at CancerCare Manitoba.
- ☐ **Information about research opportunities that are specific to a certain cancer experience.** Information about these kinds of opportunities will be shared directly with you be TheKeep.Ca team, your information will not be shared with other groups. For instance, an interview study exploring the impact of lung cancer on spouses.
- ☐ **Updates about TheKeep.Ca.** These will be shared directly with you by TheKeep.Ca team directly, your information will not be shared with other groups. These will include, but not be limited to, notices about major updates and opportunities to get involved in creating content. You will not receive these more than once a month.
- ☐ None of the above

2. I am a former or current patient/carer and would like to have my contact information shared with the patient advisor program at CCMB, as I am interested in volunteering as patient advisor. I consent to my contact information being shared with the CCMB patient advisor program.

- ◯ Yes
- ◯ No

You and your cancer experience

Please tell us a little bit about yourself.

3. What year were you born?

4. What gender do you identify as?

- ◯ Male
- ◯ Female
- ◯ Other (please specify)

5. Currently, you could be described as:

- ◯ Cancer patient
- ◯ Caregiver/supporter of a cancer patient
- ◯ Both, a cancer patient and a caregiver/supporter of someone with cancer
- ◯ None of the above

6. What year did your most recent experience with cancer begin?

7. What type of cancer are you currently experiencing, either as a patient or caregiver/supporter?

8. If you know, what is the treatment intent of the cancer?

- ◯ Cure
- ◯ To control the cancer for as long as possible, but not to cure it
- ◯ Not sure

9. What is the highest level of education you have completed?

10. Which location do you visit most often to see cancer specialist(s)?

How did you find us?

11. How did you find TheKeep.Ca?

- ◯ Google or other search engine
- ◯ Link on a website
- ◯ Social media link
- ◯ Referred by a healthcare provider
- ◯ Recommended by a friends or family member
- ◯ Other (please specify)

Contact Information

Please provide your contact information so you can be contacted about the opportunities you have selected.

12. Please provide your contact information:

First Name	
Last Name	
Email Address	
Street Number	
Street Name	
Province/State	
Postal/Zip Code	
Country	

13. How would you prefer we contact you?

- ○ Email address
- ○ Phone number
- ○ Other (please specify)

Follow-up Survey

14. TheKeep.Ca research team would like to send you a short email survey every six months to understand how this website has impacted your cancer experience. Do you consent to receiving this?

- ☐ Yes, please use the email I previously provided.
- ☐ No, do not contact me with this follow-up survey
- ☐ Yes, but please use the email address below.
