# Supplementary material for: Constructing TheKeep.Ca With Thrivers of Cancer in Manitoba, Canada, in Support of Enhancing Patient Engagement: Protocol for a Pragmatic Multimethods Study
Source: JMIR Res Protoc. 2025 Jan 29;14:e63597. doi: 10.2196/63597 (PMC11822311; doi:10.2196/63597)
Supplement: Multimedia Appendix 6 [file resprot_v14i1e63597_app6.pdf]

Things you need to know before you provide your information...

You are receiving this survey because you previously consented to receiving a follow-up survey about your experience with the TheKeep.Ca project.

By completing the following survey, you are consenting to participate in a research project to understand how TheKeep.Ca has impacted your cancer experience.

The goal of this research project is to understand how effective TheKeep.Ca website is at identifying and engaging with those that have experienced cancer. Additionally, the project aims to understand how TheKeep.Ca website impacts the experience of living with cancer.

The form being used is highly secure, compliant with the highest standards of data security. It is unlikely, but possible that a data breach may occur.

The information you provide on the form may be contribute to research and quality improvements reports. This will be done in a way where you will not be able to be identified by others.

Completing this survey should be considered strictly voluntary, and your responses, or decision to not complete the survey, will not impact your care or ability to participate in other patient engagement activities in anyway (including but not limited to research participation or work as a patient advisor).

1. When did you sign up to part of TheKeep.Ca

- ◯ within the last 2 months
- ◯ more than 2 months ago, less than 4 months ago
- ◯ more than 4 months ago, less than 6 months ago
- ◯ over 6 months ago
- ◯ not sure

2. When you signed up to be part of TheKeep.Ca, what opportunities did you consent to?

- ☐ Receiving "The Navigator" - the CCMB email newsletter for patients and informal caregivers
- ☐ Receiving information about research opportunities
- ☐ Receiving updates about TheKeep.Ca
- ☐ To having your information shared with the patient advisor program
- ☐ I can't remember
- ☐ I did not consent to any of the above when I joined TheKeep.Ca

Research Opportunities

3. In the last six months, did TheKeep.Ca assist you in finding opportunities to participate in research?

- ◯ Yes
- ◯ No

4. Was the information provided by TheKeep.Ca about these research projects helpful in helping you understand what to expect?

- ◯ Not at all, the information obtained through TheKeep.Ca was misleading
- ◯ Somewhat, I did not feel I understood what to expect from the research opportunities based on the information provided.
- ◯ Just enough information was provided to help me understand the study and what to expect, without being overwhelming
- ◯ Too much information was provided, it left me confused and/or overwhelmed

5. How would you describe the impact of participating in the research projects advertised through TheKeep.Ca?

- ◯ Positive experience - would recommend to others and
- ◯ Not positive or negative experience - might not participate again or recommend to others
- ◯ Negative experience - would not recommend to others, definitely would not repeat

6. Please share a little bit about how the experience of participating research projects identified through TheKeep.Ca has impacted you cancer experience.

[ ]

Patient Advisor Opportunities

7. Currently or in the past, have you participated in the patient advisor program at CCMB?

- ◯ Yes
- ◯ No

8. Was your decision to be involved in the patient advisor program at CCMB influenced by TheKeep.Ca?

- ◯ Yes
- ◯ No

9. In the last six months, how many patient advisor activities through CCMB have you participated in?

- ◯ 0
- ◯ 1-2
- ◯ 3-4
- ◯ 5 or more

10. How would you describe the impact of participating in the patient advisor program at CCMB?

- ◯ Positive experience - would recommend to others
- ◯ Not positive or negative experience - might not participate again or recommend to others
- ◯ Negative experience - would not recommend to others

11. Please share a little bit about how the experience of participating in patient advisor has impacted you cancer experience.

Improve TheKeep.Ca

12. Navigating TheKeep.Ca website is:

- ○ difficult and frustrating
- ○ somewhat difficult
- ○ average
- ○ the website is easy to navigate

13. The content on TheKeep.Ca is:

- ○ Not at all helpful, I find the website and/or the content frustrating
- ○ Not at all helpful, but it is not upsetting
- ○ Helpful, but it could be better
- ○ Helpful, I really enjoy the content on the website

14. What is your idea about how we could improve TheKeep.Ca?

You and your cancer experience

Please tell us a little bit about yourself.

15. What year were you born?

[ ]

16. What gender do you identify as?

- ○ Male
- ○ Female
- ○ Other (please specify)

[ ]

17. Currently, you could be described as:

- ○ Cancer patient
- ○ Caregiver/supporter of a cancer patient
- ○ Both, a cancer patient and a caregiver/supporter of someone with cancer
- ○ None of the above

18. What year did your most recent experience with cancer begin?

[ ]

19. What type of cancer are you currently experiencing, either as a patient or caregiver/supporter?

[ ]

20. If you know, what is the treatment intent of the cancer?

- ○ Cure
- ○ To control the cancer for as long as possible, but not to cure it
- ○ Not sure

21. What is the highest level of education you have completed?

[ ]

22. Which location do you visit most often to see cancer specialist(s)?

[ ]

Follow-up Survey

23. TheKeep.Ca research team would like to continue to send you a short email survey every six months to understand how this website has impacted your cancer experience and how it could be improved. Do you consent to receiving this?

- ☐ Yes
- ☐ No, do not contact me again with a follow-up survey
- ☐ No, please remove me from TheKeep.Ca database
